# ESCAschool study: trial protocol of an adaptive treatment approach for school-age children with ADHD including two randomised trials

**DOI:** 10.1186/s12888-017-1433-9

**Published:** 2017-07-24

**Authors:** Manfred Döpfner, Christopher Hautmann, Christina Dose, Tobias Banaschewski, Katja Becker, Daniel Brandeis, Martin Holtmann, Thomas Jans, Carolin Jenkner, Sabina Millenet, Tobias Renner, Marcel Romanos, Elena von Wirth

**Affiliations:** 1 Department of Child and Adolescent Psychiatry, Psychosomatics and Psychotherapy, Medical Faculty of the University of Cologne, Robert-Koch-Str. 10, 50931 Cologne, Germany; 2 School for Child and Adolescent Cognitive Behavioural Therapy (AKiP), University Hospital of Cologne, Cologne, Germany; 3 Department of Child and Adolescent Psychiatry and Psychotherapy, Central Institute of Mental Health, Medical Faculty Mannheim, Heidelberg University, Mannheim, Germany; 4 Department of Child and Adolescent Psychiatry, Psychosomatics and Psychotherapy, Medical Faculty of the Philipps-University Marburg and University Hospital Marburg, Marburg, Germany; 5 LWL-University Hospital Hamm, Ruhr-University Bochum, Hamm, Germany; 6 Department of Child and Adolescent Psychiatry, Psychosomatics and Psychotherapy, Center of Mental Health, University Hospital of Würzburg, Würzburg, Germany; 7 Clinical Trials Unit Freiburg, Medical Center – University of Freiburg, Faculty of Medicine, University of Freiburg, Freiburg, Germany; 8 Department of Child and Adolescent Psychiatry and Psychotherapy, University Hospital Tübingen, Tübingen, Germany

**Keywords:** Attention-deficit/hyperactivity disorder, School-age children, Stepped care, Adaptive treatment, Self-help, Pharmacotherapy, Behaviour therapy, Neurofeedback

## Abstract

**Background:**

The ESCAschool study addresses the treatment of school-age children with attention-deficit/hyperactivity disorder (ADHD) in a large multicentre trial. It aims to investigate three interrelated topics: (i) Clinical guidelines often recommend a stepped care approach, including different treatment strategies for children with mild to moderate and severe ADHD symptoms, respectively. However, this approach has not yet been empirically validated. (ii) Behavioural interventions and neurofeedback have been shown to be effective, but the superiority of combined treatment approaches such as medication plus behaviour therapy or medication plus neurofeedback compared to medication alone remains questionable. (iii) Growing evidence indicates that telephone-assisted self-help interventions are effective in the treatment of ADHD. However, larger randomised controlled trials (RCTs) are lacking. This report presents the ESCAschool trial protocol. In an adaptive treatment design, two RCTs and additional observational treatment arms are considered.

**Methods:**

The target sample size of ESCAschool is 521 children with ADHD. Based on their baseline ADHD symptom severity, the children will be assigned to one of two groups (mild to moderate symptom group and severe symptom group). The adaptive design includes two treatment phases (Step 1 and Step 2). According to clinical guidelines, different treatment protocols will be followed for the two severity groups. In the moderate group, the efficacy of telephone-assisted self-help for parents and teachers will be tested against waitlist control in Step 1 (RCT I). The severe group will receive pharmacotherapy combined with psychoeducation in Step 1. For both groups, treatment response will be determined after Step 1 treatment (no, partial or full response). In severe group children demonstrating partial response to medication, in Step 2, the efficacy of (1) counselling, (2) behaviour therapy and (3) neurofeedback will be tested (RCT II). All other treatment arms in Step 2 (severe group: no or full response; moderate group: no, partial or full response) are observational.

**Discussion:**

The ESCAschool trial will provide evidence-based answers to several important questions for clinical practice following a stepped care approach. The adaptive study design will also provide new insights into the effects of additional treatments in children with partial response.

**Trial registration:**

German Clinical Trials Register (DRKS) DRKS00008973. Registered 18 December 2015.

## Background

For the treatment of attention-deficit/hyperactivity disorder (ADHD), European and German clinical guidelines recommend a stepped care approach with individualised adaptive treatment strategies [1–3]. Depending on the severity of symptoms (e.g. mild to moderate, severe), different treatment protocols are recommended. Many studies have demonstrated the efficacy of single treatment options such as pharmacotherapy, behavioural interventions, neurofeedback, and self-help for parents. However, there is little knowledge about the efficacy of adaptive treatment strategies combining these interventions in the treatment process according to the patient’s needs.

Various randomised controlled clinical trials (RCTs) have demonstrated the robust efficacy of both methylphenidate and atomoxetine in the treatment of ADHD in schoolchildren. Meta-analyses indicate that the effect sizes of atomoxetine and other non-stimulant medications are significantly smaller than those of immediate-release stimulants or long-acting stimulants [4, 5].

Several meta-analyses have analysed the efficacy of behavioural interventions including parent management training and school-based interventions. For psychosocial interventions, the analyses found moderate [6] to large [7] effect sizes. Recent meta-analyses revealed effects on core ADHD symptoms in unblinded but not in blinded ratings [8, 9]. However, the blinded ratings used may not be valid for the assessment of the effects of parent training at home. Moreover, a higher efficacy of combined behavioural and pharmacological treatment compared to pharmacotherapy alone remains questionable [6, 10, 11]. This may be explained, at least in part, by the high efficacy of pharmacotherapy. In patients showing a strong treatment response to pharmacotherapy, there is only little room for further improvement, whereas in patients with a partial or no response to medication, additional effects of psychotherapy or other psychosocial interventions seem to be more likely.

Although pharmacotherapy and behaviour therapy are established treatment options, they are intense, time-consuming and may have side effects [12]. Waiting lists for outpatient treatments are common in most European countries, as there is an imbalance between the availability of child psychiatric and psychotherapeutic services and patients’ demand. Therefore, there is a need for low-threshold therapeutic services that are easy to disseminate. Reviews indicate growing evidence for the efficacy of telephone-assisted self-help interventions for parents of children with ADHD [13, 14]. However, there is a lack of larger RCTs and of trials evaluating this intervention as an initial low-threshold type of treatment in patients with less severe ADHD symptoms. In a recent study not covered by the aforementioned reviews, telephone-assisted self-help for parents of 8–12-year-old children with ADHD proved to be more effective than treatment as usual [15]. Our own research has demonstrated the effects of telephone-assisted self-help for parents of preschool children with ADHD and other externalising behaviour problems [16, 17]. Moreover, a large observational study employing a self-help programme for parents demonstrated symptom reduction in school-age children with ADHD [18], and a recent study demonstrated additional effects of telephone-assisted self-help on externalising symptoms in school-age children with ADHD and persisting functional impairment despite methylphenidate treatment [19]. A further recent study indicated that both behaviourally oriented and non-directive-oriented telephone-assisted self-help interventions may be effective in the treatment of externalising behaviour problems [20].

Neurofeedback has gained some empirical support in recent years. Meta-analytic findings following a rigorous methodological approach found small to moderate significant treatment effects of neurofeedback on ADHD scores from unblinded informants [9, 21]. Although the effect of neurofeedback was non-significant in blinded assessments and in trials with active/sham controls, somewhat larger and significant effects were found in the few studies using standard neurofeedback protocols [21].

Adaptive interventions customize the individual treatment during the therapy process to the patient’s needs and responses to previous treatments. This may include a change of the intensity of an already existing intervention, a change of the type of intervention, or a combination of the previous treatment with other forms of therapy. For the treatment of ADHD, adaptive interventions have barely been investigated. Nevertheless, some growing interest over the years can be discerned [22–28]. For example, a study examining adaptive multimodal treatment in children with ADHD detected additive effects of pharmacotherapy and psychotherapy in patients who responded only partially to medication [23].

The primary objective of the study *Evidence-Based, Stepped Care of ADHD in School-Age Children Between 6 and 11 Years* (ESCAschool) is to assess the efficacy of a stepped care approach involving individually tailored adaptive treatment strategies. The secondary objective is to examine the predictability of treatment response by psychological (e.g. child temperament, parental psychopathology) and biological domains (e.g. electroencephalography). Interventions included in the trial are (i) telephone-assisted self-help for parents and teachers, (ii) individual behaviour therapy, (iii) pharmacotherapy (plus psychoeducation or counselling), and (iv) neurofeedback. As recommended by European and German clinical guidelines [1, 3], different treatment protocols will be followed for children with mild to moderate versus severe ADHD. For all patients and their families, the intervention will be divided into two treatment periods (Step 1, Step 2), with treatment in Step 2 depending on the patient’s response to the treatment in Step 1 (no, partial and full response). This report presents the ESCAschool trial protocol (version 4 from 22 December 2016) and has been conceived under consideration of the SPIRIT guidelines [29, 30].

ESCAschool is part of the research consortium ESCAlife (http://www.esca-life.org/). The primary aim of the umbrella project is to investigate adaptive interventions for patients with ADHD from preschool age to adulthood. Besides ESCAschool, ESCAlife encompasses the ESCApreschool study for preschool children aged 3 to 6 years, the ESCAadol study for adolescents aged 12 to 15 years and the ESCAlate study for adolescents and adults aged 16 and 45 years.

## Methods/design

### Sites

This is a multisite trial with nine study centres located in Germany (Cologne, Essen, Göttingen, Hamm, Mainz, Mannheim, Marburg, Würzburg, Tübingen). If required, additional centres will be included. The coordinating centre is the Department of Child and Adolescent Psychiatry, Psychosomatics and Psychotherapy, Medical Faculty of the University of Cologne and has the main responsibility for the trial protocol, the content and extent of the treatment as well as supervision. One exception is the treatment planning for neurofeedback which is primarily organized by Hamm and Mannheim. In general patients are treated locally at the respective study centres with the exception of telephone-assisted self-help for parents and teachers which is centrally provided and exclusively conducted by Cologne. Responsibility for monitoring, data management, biometry and project coordination has the Clinical Trials Unit at the University Medical Centre Freiburg.

### Inclusion and exclusion criteria

Families have to meet the following inclusion criteria: (i) child age 6;0 to 11;11 years; (ii) child attendance of school (including special schools); (iii) child meeting criteria for ADHD diagnosis according to the DSM-5 [31]; (iv) existence of informed consent of both parents or guardians and assent of the child.

Exclusion criteria are: (i) child intelligence quotient (IQ) below average (IQ < 80); (ii) child clinical diagnosis of a pervasive developmental disorder, schizophrenia, bipolar disorder, severe depressive episode, epilepsy or heart disease; (iii) insufficient German language or reading skills of the parent with primary treatment involvement; (iv) current or planned intensive behaviour therapy for child ADHD or oppositional behaviour on a weekly basis; (v) known non-response of the child to all standard ADHD medication (methylphenidate, dexamphetamine, atomoxetine); (vi) psychotropic medication of the child other than for the treatment of ADHD, or neuroleptic medication other than for the treatment of disturbances of impulse control.

### Study design

**Fig. 1:**
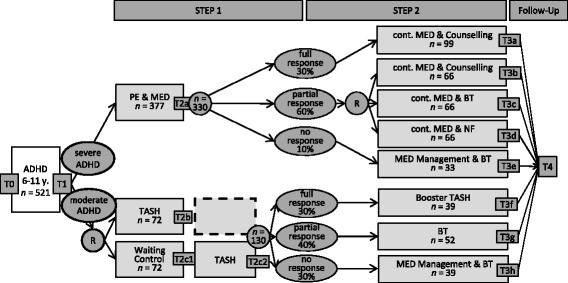
Study design of the ESCAschool study testing a stepped care approach and adaptive intervention strategies for children with ADHD including different treatment protocols for children with mild to moderate symptoms and children with severe symptoms. The percentages in the ovals for the response categories indicate expected response rates to the treatment in Step 1. T0 to T4 = assessment points; R = randomisation; PE = psychoeducation; MED = ADHD medication treatment; TASH = telephone-assisted self-help for parents; BT = behaviour therapy; NF = neurofeedback; SH = self-help; *n* = planned/estimated sample size

The trial design is described in Fig. 1. Children who are eligible for the study will be categorised into a group with mild to moderate ADHD symptoms and a group with severe symptoms based on a clinician-administered, structured interview with the parent according to DSM-5 criteria. Both groups will undergo two treatment phases; the first (Step 1) will take 3 months (for waiting-list patients with mild to moderate symptoms, this period will last for 6 months) and the second (Step 2) will last for 6 months.

For children with severe ADHD, Step 1 treatment will comprise psychoeducation and ADHD medication treatment (PE & MED; see Fig. 1). The interventions in Step 2 will be chosen depending on the response to the treatment in Step 1. Step 1 full responders will receive continued medication plus counselling (Cont. MED & Counselling). Partial responders will be randomised to one of three treatment arms: (i) continued medication plus counselling (Cont. MED & Counselling), which is identical to the treatment of full responders, (ii) continued medication plus behaviour therapy (Cont. MED & BT), or (iii) continued medication plus neurofeedback (Cont. MED & NF). Step 1 non-responders will be referred to alternative pharmacotherapy according to treatment guidelines and will additionally receive behaviour therapy (MED Management & BT).

Families of children with mild to moderate ADHD symptoms will be randomised in Step 1 either to telephone-assisted self-help (TASH) for parents and teachers or to a waitlist control group (Waiting Control; see Fig. 1). Waiting Control families will receive TASH after a 3-month waiting period (for these patients, Step 1 will last for 6 months). In Step 2, for children with mild to moderate ADHD symptoms, the following treatments will be delivered depending on the response to the treatment in Step 1: Step 1 full responders will receive booster sessions of telephone-assisted self-help for parents and, if desired, for teachers (Booster Self-Help). Step 1 partial responders will receive behaviour therapy (BT) and Step 1 non-responders will receive pharmacotherapy plus behaviour therapy (MED Management & BT).

### Definition of symptom severity at baseline

At baseline, children will be assigned to one of two ADHD symptom severity groups based on a clinical rating: (a) a group with mild to moderate symptoms and (b) a group with severe symptoms. Severe group children have to meet both of the following criteria: (i) a mean item score ≥ 2 (possible range 0–3) on the inattentiveness scale and/or the hyperactivity/impulsiveness scale of a clinician-administered, structured interview assessing DSM-5 criteria for ADHD (*Interviewleitfaden mit Diagnose-Checkliste für Aufmerksamkeitsdefizit−/Hyperaktivitätsstörungen* [DCL-ADHS]) [32], (ii) a rating ≥ 5 on the severity scale (range 1–7) of the *Clinical Global Impressions* (CGI) [33]. All children who are eligible for the study, but who do not meet these criteria, will be assigned to the moderate group.

### Definition of treatment response and expected response rates

After Step 1, the treatment response of all children will be determined by a clinical rating. Three response groups will be distinguished: (a) patients showing full treatment response, (b) patients showing partial treatment response and (c) patients with no treatment response.

Full responders have to fulfil all of the following criteria: (i) mean item score ≤ 1 on the inattentiveness scale of the DCL-ADHS; (ii) mean item score ≤ 1 on the hyperactivity/impulsiveness scale of the DCL-ADHS; (iii) CGI severity scale score < 3.

To be classified as a partial responder, a patient has to fulfil the following criteria: (i) a reduction of the DCL-ADHS total score by at least 20% compared to baseline; (ii) a mean item score > 1 on the inattentiveness scale of the DCL-ADHS or a mean item score > 1 on the hyperactivity/impulsiveness scale of the DCL-ADHS or a score ≥ 3 on the CGI severity scale. The second criterion serves to indicate that there are still clinically meaningful ADHD symptoms or a meaningful impairment in these patients.

Non-responders are defined as follows: (i) Reduction in DCL-ADHS total scale is less than 20% compared to baseline. (ii) The second criterion for non-responders is identical to criterion ii for partial responders.

It is hypothesised that 30% of the children in the severe group will be full responders, 60% will be partial responders and 10% non-responders. These estimates are derived from the response rates in trials on the efficacy of pharmacotherapy [4, 5]. Based on previous trials on self-help interventions for parents of children with ADHD [13, 34], 30% of the patients in the moderate group are estimated to be full responders, 40% to be partial responders, and 30% to be non-responders.

### Trial interventions

The intervention study is based on four major therapy approaches: pharmacotherapy (plus psychoeducation or counselling), behaviour therapy, neurofeedback, telephone-assisted self-help. These interventions will be offered either as single treatments or in combination.

#### Pharmacotherapy (plus psychoeducation or counselling)

Pharmacotherapy will be conducted by physicians of the study centres or by local physicians with primary responsibility for child care. Practitioners will be advised to follow treatment guidelines for pharmacotherapy of ADHD, but there are no study-related restrictions regarding their choice of substance class or dosage. However, if Step 1 therapy starts with a first-line stimulant medication that proves to be insufficient after titration (e.g. immediate-release methylphenidate), practitioners will be asked to reconsider another stimulant medication for Step 1 treatment (e.g. sustained-release methylphenidate, dexamfetamine, lisdexamfetamine) and to preserve a change in the drug class to Step 2 treatment (e.g. non-stimulant) if this procedure is medically acceptable.

For pharmacological treatment, the number of visits will depend on the patient’s individual needs (about three visits planned in Step 1 and about four in Step 2). For psychoeducation or counselling, four additional sessions are scheduled.

#### Behaviour therapy

Individually tailored behaviour therapy will encompass (i) parent management training, including parent-child interaction training, (ii) child-focused interventions, especially organisational skills training, (iii) teacher-focused interventions, including psychoeducation and behavioural interventions at school. Twenty sessions are planned on a weekly basis. The treatment is primarily based on the German treatment manual *Therapieprogramm für Kinder mit hyperkinetischem und oppositionellem Problemverhalten* (THOP), which includes parent-, child- and teacher-focused interventions [35]. The efficacy of this intervention has been demonstrated in several clinical trials [23, 24, 27, 36, 37]. In addition, child-focused organisational and planning skills training interventions based on the German *Therapieprogramm zur Steigerung von Organisationsfähigkeit, Konzentration und Impulskontrolle bei Kindern mit ADHS* (THOKI-ADHS) will be included [38]. This manual describes interventions evaluated by Abikoff et al. [39] and Langberg et al. [40]. For each patient, an individual treatment plan will be established together with the supervisor after the fifth treatment session. The treatment will be conducted by clinical therapists who will be trained in the specific interventions in a two-day workshop.

#### Neurofeedback

Neurofeedback will be conducted following a slightly modified protocol of a previous controlled study [41]. In ESCAschool, participants will receive 25 sessions of neurofeedback of slow cortical potentials (SCPs). Each session will last for approximately 60 min, including time needed for electrode montage as well as four 10-min blocks of feedback. Each block consists of 40 trials and each trial is composed of a baseline phase (2 s) and a feedback phase (8 s). If patients are successful for at least 2 s in total during the second half of the feedback phase, a sun is shown as positive reinforcement. The training protocol prompts either negative potential or positive potential shifts compared to the baseline [42]. Within the first 12 training sessions, negativity and positivity are trained in randomised succession in a 50:50 ratio. The ratio is changed to 60:40 within the last 13 sessions. During each session, feedback is given in blocks 1 and 3. To enable the transfer of self-regulation skills to everyday life, participants perform the second and fourth blocks of each session without continuous feedback (transfer trials). Participants are able to earn a certain amount of tokens for taking part and good cooperation. To strengthen the transfer, every session is followed by a short transfer exercise before doing some homework in the lab.

Electroencephalography (EEG) is recorded and fed back with a multichannel amplifier (THERA PRAX®, neuroConn GmbH, Ilmenau, Germany). The EEG electrode is placed at Cz, referenced against the mastoid behind the right ear. Four electrodes are used to record the vertical and horizontal electrooculogram (EOG) and one electrode behind the right ear is used as ground. Each study centre will be guided by a detailed manual to ensure equal handling of participants and devices.

#### Telephone-assisted self-help

The behaviour therapy-oriented telephone-assisted self-help programme will address both the parents and the teachers of the children. It consists of self-help booklets on ADHD and behaviour modification techniques plus additional telephone consultations. The parents and teachers will receive the booklets by mail and will be asked to read them and to implement the interventions described therein. Furthermore, the telephone consultations will serve to help the parents or teachers to apply the interventions to the child’s specific problem behaviours. The parent programme includes eight booklets [43] and ten 20–30-min telephone consultations carried out approximately once a week. The booklets for parents are revised versions of booklets used in earlier trials, which have already demonstrated the efficacy of telephone-assisted self-help [16, 17, 19, 20], and deal with the following issues: (a) definition of individual problem behaviours of the child which the parents wish to address during the intervention, (b) psychoeducation about ADHD including information about the aetiology of ADHD, comorbidities, and different treatment options, (c) encouragement of positive parent-child interactions, (d) implementation of family rules, (e) effective commands and appropriate positive consequences for following rules, (f) appropriate negative consequences for breaking rules, (g) reward systems, and (h) development of a daily structure, leisure activities of the child (e.g. use of media), and stress reduction techniques for parents. The teacher programme is composed of four self-help booklets [44] and four telephone consultations of up to 60 min each. The contents of the booklets are similar to those of the parent booklets, but some sections are modified to fit better to the school environment. The participation of the teacher is optional; only if both the parents and the teacher agree to participation the teacher will be contacted.

If children are full responders to the three-month self-help intervention in Step 1, two additional telephone consultations for the parents and, where appropriate, one additional telephone consultation for the teacher will be provided (Booster Self-Help).

### Treatment fidelity

Treatment fidelity will be assured by (i) training in manualised treatment procedures (except pharmacotherapy), (ii) a structured protocol completed by therapists after each session, and (iii) supervision of behaviour therapy and telephone-assisted self-help by senior supervisors, either face-to-face or by telephone. Behaviour therapies will be supervised after treatment sessions 5, 10 and 15, including a review of at least two videotaped sessions. Supervision for self-help will be scheduled on a weekly basis and may comprise audiotaped telephone calls whenever required.

### Informants

The following informants will be considered for assessment: unblinded clinician, blinded clinician, parent, partner, and teacher. The unblinded clinician is a member of the project staff, is involved in diagnostics or in therapy and may be aware of the treatment condition and the time of the assessment, although efforts are taken to blind the raters. The blinded clinician is also a member of the project staff, is involved only in diagnostics and is aware of neither the treatment condition nor the time of the assessment. His or her rating is based on videotaped records of parent interviews conducted by the unblinded clinician. The parent may be the biological parent or the guardian of the child and is involved in the treatment. The partner is the second parent or guardian and is generally not primarily involved in the treatment (only completes the baseline assessment T1). The teacher is the child’s schoolteacher, preferably the class teacher with the main responsibility for the child’s school routine.

### Measurements – Main assessment points and therapy process data

During the trial, data will be collected (a) at several main assessment points before and after the interventions as well as (b) during the therapy process. The main assessment points (T0/T1, T2, T3, T4) will take place according to a specified schedule (see Fig. 1). The first two measurements (T0/T1) serve to check the inclusion and exclusion criteria and to collect baseline data. The third measurement (T2) will take place after Step 1 treatment (note that there are two T2 assessments for families randomised to Waiting Control in the moderate group: one after the three-month waiting period and one after the telephone-assisted self-help which they receive afterwards; see Fig. 1). The fourth measurement (T3) will be conducted immediately after Step 2 treatment. The follow-up measurement (T4) will occur three months after the end of Step 2 treatment. In addition to the main assessment points, the physicians and therapists who conduct the intervention will provide ratings during the therapy process after each session (e.g., adherence and integrity).

### Primary and secondary outcomes

Unless otherwise stated, all primary and secondary outcome measures will be assessed at all main assessment points (T0/T1, T2, T3, T4).

#### Primary outcome – Blinded clinician

The primary outcome is the blinded clinician-rated ADHD score assessed by the total scale (18 items) of the German *Diagnose-Checkliste für Aufmerksamkeitsdefizit−/Hyperaktivitätsstörung*en (DCL-ADHS) based on the German structured interview *Interviewleitfaden für Externale Störungen* (ILF-EXTERNAL) conducted with the parent [32]. The items reflect the criteria for ADHD according to the DSM-5 and ICD-10 [45].

#### Secondary outcomes – Blinded clinician

Impairment due to ADHD symptoms will be rated on the respective scale (5 items) of the DCL-ADHS. The German *Diagnose-Checkliste für Störungen des Sozialverhaltens* (DCL-SSV) serves to assess symptoms of oppositional defiant disorder (ODD; 8 items), symptoms of disruptive mood dysregulation disorder (DMDD; 2 items), core symptoms of conduct disorder (CD; 7 items), characteristics of limited prosocial emotions (11 items) and impairment due to disruptive behaviour problems (5 items) according to the ICD-10 and DSM-5 [32]. The DCL-SSV is based on ILF-EXTERNAL conducted with the parent. A further secondary outcome rated by the blinded clinician is the severity scale (1 item) of the *Clinical Global Impressions* (CGI) [33].

#### Secondary outcomes – Unblinded clinician

All instruments available in blinded clinical rating (DCL-ADHS, DCL-SSV and CGI severity) will have been previously rated and videotaped by the unblinded clinician involved in assessment. In addition, the unblinded clinician will complete the improvement scale (1 item) of the CGI and rate adherence to medication (if applicable), psychological side effects of the therapy (6 self-developed items; assessed at T2 and T3) and additional professional interventions received by the families besides the study treatment (assessed at T2, T3 and T4). Furthermore, therapists will rate their treatment satisfaction (8 self-developed items; assessed at T2 and T3).

#### Secondary outcomes – Parent

The parent will complete the German *Fremdbeurteilungsbogen für Aufmerksamkeitsdefizit−/Hyperaktivitätstörungen* (FBB-ADHS), which assesses ADHD symptoms (18 items) and related impairment (5 items) [32] as well as the German *Fremdbeurteilungsbogen für Störungen des Sozialverhaltens* (FBB-SSV), which captures ODD symptoms (8 items), DMDD symptoms (3 items), CD symptoms (6 items), characteristics of limited prosocial emotions (11 items), and impairment due to disruptive behaviour problems (5 items) [32]. The FBB-ADHS and FBB-SSV both consider criteria according to the DSM-5 and ICD-10. Further measures are the internalizing problems scale (32 items) of the German translation of the *Child Behavior Checklist for Ages 6–18* (CBCL/6–18R) [46, 47], the German version of the *Weiss Functional Impairment Rating Scale – Parent Report* (WFIRS-P) [48, 49], which measures functional impairment, and the KIDSCREEN-10 Index [50] to assess child quality of life (10 items). Parenting-related measures comprise the total scale (27 items) of the German questionnaire *Verhalten in Risikosituationen* (VER) to assess the perceived ability to solve difficult parenting situations [51], the total scale (13 items) of the German questionnaire *Fragen zum Erziehungsverhalten* (FZEV) to measure positive, reinforcing and promotive parenting practices [51], and a short version of the negative scale (13 items) of the German *Fragebogen zum positiven und negativen Erziehungsverhalten* (FPNE) to assess dysfunctional parenting practices [52]. Satisfaction with Step 1 treatment (8 self-developed items) will be assessed at T2 and satisfaction with Step 2 treatment at T3.

#### Secondary outcomes – Teacher

Teachers will rate the same items of the FBB-ADHS and FBB-SSV as those rated by the parent.

#### Secondary outcome – Biological

For a subgroup of children, EEG will be measured before and immediately after Step 2 treatment (T2 and T3). EEG will only be measured in children who receive neurofeedback or behaviour therapy as part of the treatment in Step 2. EEG outcome parameters are the resting theta activity (hypothesised reduction) and the contingent negative variation (CNV) amplitude (hypothesised increase).

### Predictors of treatment response

The secondary objective of the ESCAschool study is the prediction of treatment response. This concerns variables assessed prior to Step 1 (T0/T1) and Step 2 treatment (T2) as well as therapy process data (Step 1, Step 2).

#### Predictors – Psychological data

Psychological data will be collected from (a) the unblinded clinician, (b) the parent, (c) the partner, and (d) in the form of test diagnostics.

##### Unblinded clinician

The following variables will be assessed by a clinical interview with the parent during diagnostic assessment at T0/T1: sociodemographic data of the child and the parent (e.g. child age, education of the parent or guardian), early child development (6 self-developed items) and temperament (13 self-developed items), life events (14 self-developed items), and the *Family Adversity Index* (FAI) adopted from the German *Mannheimer Elterninterview* [53]. The clinical checklist *Diagnose-Checkliste zum Screening psychischer Störungen* (DCL-SCREEN) will be applied to assess comorbid symptoms of depression (7 items), autism (4 items), anxiety (10 items), other neurodevelopmental disorders (6 items), obsessive-compulsive disorder (2 items) and tic disorders (1 item) [32]. Based on a modified questionnaire by Piacentini et al. [54], the therapists will additionally report their expectation of treatment benefit (3 items) for a family assigned to a particular treatment arm (rated at T0/T1 before Step 1 treatment and at T2 before Step 2 treatment). Furthermore, every therapy session of Step 1 and Step 2 treatment will be rated with respect to treatment integrity (13 self-developed items), treatment adherence of the client (10 items; for TASH only 8 items), and current ADHD symptoms of the child (4 items; shortened version of the German *ADHS-Bogen*) [55].

##### Parent

The following domains will be assessed by ratings of parents at T0/T1: child irritability (7 items) by the *Affective Reactivity Index* (ARI) [56], child personality (novelty seeking, harm avoidance, reward dependence, persistence, self-directedness, cooperativeness, self-transcendence) by the German *Junior Temperament und Charakter Inventar* [57] in the version for children aged 7 to 11 years (JTCI 7–11 R; 86 items), severity of social deficits in the autism spectrum by a short version (16 items) of the German translation of the *Social Responsiveness Scale* (SRS) [58–60]. Moreover, parents will complete the anger control scale of the German *Elternfragebogen zum Umgang mit Ärger* (FB-Ä) [61], which is a modified version of the 12-item form of the *Aggression Questionnaire* [62, 63] to measure parental aggression. Depression, anxiety and stress will be assessed by the German 21-item version [64, 65] of the *Depression Anxiety Stress Scales* (DASS) [66] and parental ADHD by the German *ADHS Selbstbeurteilungsskala* (ADHS-SB) [67], which was adapted to the DSM-5 for use in this study.

##### Partner

The parent with no primary involvement into the treatment will complete the FB-Ä to assess parental aggression and the ADHS-SB to measure parental ADHD at the beginning of the study (T0/T1).

##### Test diagnostics

At the beginning of the study (T0/T1), the following test diagnostics will be applied: IQ will be assessed using the total score of the German translation of the *Wechsler Nonverbal Scale of Ability* [68, 69]. Further neuropsychological testing will encompass a variant of the *Continuous Performance Task* (CPT-OX) [70] to assess sustained and selective attention. A modified version of the *Monetary Incentive Delay* (MID) task [71, 72] will be used to investigate reward anticipation and reward feedback, and a *Stop-Signal Task* (SST) [72, 73] will be conducted to study inhibitory control. For children receiving ADHD-related pharmacotherapy prior to the start of the study, IQ will be tested under medication and neuropsychological testing will be conducted without medication.

#### Predictors – Biological data

The following imaging methods will be used to assess biological predictors of treatment response in a sub-project called ESCAbrain: EEG, magnetic resonance imaging (MRI) and transcranial sonography (TCS). TCS will be conducted in all patients and is not formally bound to any of the main assessment points (T0/T1 to T4). EEG and MRI are limited to patients who receive neurofeedback or behaviour therapy as part of their treatment in Step 2 (see Fig. 1). MRI assessments will be conducted prior to the beginning of Step 2 treatment (T2), whereas EEG assessments will be performed prior to and after Step 2 treatments. Furthermore, blood samples (and/or buccal swabs) will be collected before Step 1 (T0/T1) and after Step 2 treatment (T3) to determine predictive genetic and epigenetic patterns.

##### EEG

The EEG-based predictors consist of the frequency profile at rest (spontaneous theta band and alpha band activity) and the strength of preparatory cognitive activity (CNV amplitude) measured during a cued continuous performance task (CPT), which represent promising biological markers of ADHD and have been shown to explain nearly 30% of the variability in behavioural improvement following neurofeedback treatment [74].

##### MRI

The MRI-based predictors consist of the integrity (fractional anisotropy) of the fronto-striatal connection, and of volumetric grey matter density of the implicated dorsolateral-prefrontal and striatal regions, which also represent promising biological ADHD markers [75, 76].

##### TCS

The TCS-based predictor is the extent of the echogenic region of the substantia nigra and has been identified as a potential biological ADHD marker [77].

### Planned sample size and power calculations

Based on power calculations, 521 children have to be included in the study. The entire stepped care design is primarily powered for the two included RCTs. RCT I concerns the moderate group, with randomisation for Step 1 treatment, while RCT II addresses partial responders of the severe group, with randomisation for Step 2 treatment.

For partial responders of the severe group, an effect size of Cohen’s *d* = 0.6 is hypothesised regarding the comparison of Cont. MED & Counselling (control) with Cont. MED & BT, and also for the comparison of Cont. MED & Counselling with Cont. MED & NF. With 59 patients per arm available for assessment after Step 2 treatment (T3), a one-way analysis of variance to compare all three arms at a significance level of 5% will have 92% power (software: NQuery Advisor Version 7.0), and a two-sided *t*-test at a significance level of 5% will have 89.8% power (software: STPLAN Version 4.3) for two-arm comparisons to detect a difference when the true effect size is *d* = 0.6. Hence, a closed testing procedure comparing two arms only if significant differences are detected between the three arms will yield at least 82.6% power for the two-arm comparisons of interventions to control. To account for the possibility that about 10% of patients will have incomplete data for the assessment after Step 2 treatment (T3), in total, 198 partial responders should be randomised after T2 assessment for Step 2 treatment. Assuming that 60% of the patients are partial responders after Step 1 treatment (T2), and 10% of patients drop out from baseline (T0/T1) to T2, about 377 patients with severe ADHD will have to be included at T0/T1 in order to achieve the target number of 198 randomisations.

Regarding treatment arms with randomisation in the moderate group, for the comparison of TASH with Waiting Control, an effect size of *d* = 0.5 is expected. Using a two-sided *t*-test with a power of 80% at a significance level of 5%, 64 patients with non-missing data per group are required to detect a difference when the true effect size is *d* = 0.5. Assuming that 10% of the families will have incomplete data for the assessment after Step 1 treatment (T2), in total, 144 patients with moderate ADHD should be randomised after T0/T1 assessment for Step 1.

### Compliance and rate of loss to follow-up

In our own pharmacotherapeutic trials as well as the trials on self-help and those on behaviour therapy or neurofeedback with similar treatment durations, losses to follow-up were in the range of 6 to 10% [17, 23, 34, 78].

### Statistical analysis

The primary statistical analyses will be intention-to-treat analyses; that is, all randomised patients will be analysed according to their allocated arms irrespective of whether they refused or discontinued the treatment or whether other protocol violations are revealed.

For the moderate group, the difference between TASH and Waiting Control at T2 and in the severe group for partial responders the difference between the three randomised treatments in Step 2 at T3 will be evaluated in separate linear regression models including treatment, centre, and the respective baseline value as predictors. Further covariates predictive of missingness will be included based on a pre-specified selection strategy to correct for potential bias arising from missing data. Under the assumption of missing at random (MAR), a complete case analysis will be performed. As sensitivity analysis, a worst-case analysis will be performed, assigning the best possible outcome to missing values in the control group and the worst possible outcome to those in the experimental group(s). If this extreme analysis is still favourable, then it can be confidently concluded that the results are robust to the handling of missing data. The significance test for the primary treatment comparison in the moderate group will be based on least-squares means and will be presented with two-sided 95% confidence intervals. For the severe group, a closed test procedure will be applied: First, the null-hypothesis of equal means in the three arms will be tested at a significance level of 5%. If it can be rejected, the three pairwise treatment comparisons will be carried out based on two-sided 95% confidence intervals. This multiple test procedure assures control of the multiple type I error rate of 5%.

Besides the primary statistical investigation, several secondary analyses will be conducted. These include the analysis of change during Step 1 and Step 2 treatment separately for all study arms. Further, for certain subgroups, within-group comparisons will be conducted and change during Step 1 will be contrasted with change during Step 2 (i.e., test whether change during Step 1 and Step 2 differs for a particular subgroup). These within-group comparisons concern full responders and non-responders in the severe group as well as all response types of the moderate group. Finally, predictors and moderators of treatment response will be analysed [79].

### Patient registration and randomisation

Patients will be centrally registered at the Clinical Trials Unit Freiburg. To guarantee concealment, central randomisation at Step 1 and Step 2 will be performed. Randomisation forms that are completed at the study centres will be sent by fax to the Clinical Trials Unit Freiburg. The study centre will be subsequently informed about the randomised treatment arm by fax. If the details on the randomisation fax appear to be incomplete or implausible, the Clinical Trials Unit Freiburg will send a query fax to the investigator for clarification.

The randomization code will be generated by the Clinical Trials Unit Freiburg to ensure that treatment assignment is unbiased and concealed from patients and investigator staff. Randomization will be performed in blocks of variable length in a ratio of 1:1. The block lengths will be documented separately and will not be disclosed to the center. The randomization code will be produced by validated programs based on the statistical analysis system.

### Data protection

Data will be entered and processed as soon as the signed informed consent is available. All data relevant for the trial will be typed into an electronic remote data entry system (RDE-LIGHT) from all centres. The Clinical Trials Unit Freiburg will provide the data entry system. Patient data will be registered pseudonymously. RDE-LIGHT uses built-in security features to encrypt all data for transmission in both directions, preventing unauthorised access to confidential participant information. Access to the system will be controlled by individually assigned user identification codes and passwords, made available only to authorised personnel.

Each patient will be identified with a study-specific patient number, which will be allocated as soon as the patient is included in the study. The patient number includes information about the centre (centre-specific numbers will be allocated before trial start) as well as a patient-specific number.

The Clinical Trials Unit Freiburg will process data through personnel specifically trained for the study, who will work according to the standard operating procedures of the study centres. Legal regulations for data protection will be fulfilled.

### Data monitoring

Monitoring is performed by the Clinical Trial Unit Freiburg. The study centres will undergo monitoring visits before, during and after the clinical trial. Prior to the trial, a pre-trial visit by phone and face-to-face group trainings are conducted in order to introduce the study centres and to train staff members in the implementation of treatments and clinical interviews. During the trial, the monitor will visit the site regularly depending on the recruitment rate and quality of the data. Details about the procedures are provided in a monitoring manual.

### Stopping rules

#### Stopping rules for an individual patient

One (or more) of the following circumstances will result in an early study termination of single subjects (these trial subjects will be rated as drop-out): (i) withdrawal of informed consent of parents/guardians, (ii) withdrawal of assent of the patient, (iii) unwillingness to further participate in the trial, (iv) need for inpatient treatment or other reasons affecting the patient’s well-being in the case of continued trial participation, (v) need for a different kind of treatment for health reasons according to the judgement of the attending physician.

#### Global stopping rules

One (or more) of the following circumstances will result in an early termination of the entire trial or in the closing of a single centre: (i) emergence of data leading to a revision of the risk-benefit ratio. (ii) Participating centres will be closed in the case of ongoing failure of recruitment or repeated violations of the study protocol or of standard good clinical practice rules.

However, prior to closing a centre, in the case of falling below the expected recruitment rate, compensation by existing centres is intended (if necessary, new study centres will be opened). Agreement between principal investigator, site investigators, responsible ethics committee and the Clinical Trials Unit Freiburg is intended.

### Legal and ethical foundation

Before trial start, all relevant documents have been submitted to the ethics committee responsible for the respective participating centre. The primary vote of the study has been obtained from the ethics committee of the medical faculty of the University of Cologne.

All relevant changes to the trial protocol have to be reported to the ethics committee. For changes to the trial protocol that are formal in nature and include relevant changes for trial subjects, the ethics committee has to vote anew. Patients/trial subjects will have to be informed about changes in the conditions of the study if necessary. The ethics committee will have to be informed immediately about complications and severe adverse events during the project.

### Risk-benefit considerations

The essential study-related procedures will take place in routine care or complement routine care. The outpatient unit will ensure the availability of professional counselling (including treatment recommendations) even if the patient does not participate in the study.

The efficacy of the guided self-help intervention for participants in the moderate group (Step 1) has been confirmed in several studies. A waiting time of 3 months is justifiable compared to the usual waiting time for treatment in routine care. Waiting Control will receive the guided self-help intervention after the waiting period.

There are no known risks for the guided self-help intervention, behaviour therapy, or neurofeedback. Possible side effects of the pharmacological treatment have been investigated intensively and will be monitored in regular patient contacts. Medication will be administered in routine care.

Patients might be able to draw direct personal benefit from participation in the study (e.g. through a more intensive support) and future patients might benefit through the incorporation of the study results into guideline recommendations. Thus, treatment recommendations will be more valid. Overall, the benefits of the study exceed any possible risks.

## Discussion

Clinical guidelines recommend a stepped care approach with an individualised adaptive treatment. However, this approach has not yet been empirically validated. The main goal of the ESCAschool study is to assess the efficacy of a stepped care approach in children with ADHD aged 6 to 11 years and to investigate predictors of treatment outcome. Different stepped care strategies for children with mild to moderate versus severe ADHD will be investigated.

Two RCTs are implemented in the design to assess the efficacy of interventions at crucial points during the therapy process. The first RCT concerns the efficacy of telephone-assisted self-help for parents and teachers and will be performed in children with mild to moderate ADHD, since there is a lack of trials evaluating this intervention as an initial low-threshold type of treatment in patients with less severe ADHD symptoms.

The additional effects of behaviour therapy and neurofeedback in patients with severe ADHD and with partial response to medication will be evaluated in the second RCT, because the supplementary effects of these interventions have not yet been sufficiently investigated for this particular subgroup of patients. The aim is to investigate in more detail which treatment approach is best suited for children who are already treated with medication but still show scope for symptom improvement.

The results will provide information about the treatment of school-age children with ADHD and will help to develop usable, potentially more cost-effective, individualised stepped care approaches. The evaluation of predictors of treatment response will help to identify indications for treatments.

## Abbreviations

Booster Self-Help: Step 2 treatment arm for full responders in the moderate group receiving booster sessions of telephone-assisted self-help for parents and teachers
BT: Step 2 treatment arm for partial responders in the moderate group receiving behaviour therapy.
Cont. MED & BT: Step 2 treatment arm for partial responders in the severe group receiving continued ADHD medication treatment and behaviour therapy.
Cont. MED & Counselling: Step 2 treatment arm for partial responders and full responders in the severe group receiving continued ADHD medication treatment plus counselling.
Cont. MED & NF: Step 2 treatment arm for partial responders in the severe group receiving continued ADHD medication treatment and neurofeedback in Step 2.
MED Management & BT: Step 2 treatment arm for non-responders in the moderate group and in the severe group receiving (alternative) pharmacotherapy plus behaviour therapy in Step 2.
PE & MED: Step 1 treatment arm of the severe group receiving psychoeducation plus ADHD medication treatment.
Step 1: First of two treatment periods with a duration of 3 months; for Waiting Control condition of the moderate group, Step 1 takes 6 months, because after the waiting period participants receive the self-help programme.
Step 2: Second of two treatment periods with a duration of 6 months.
T0/T1: Screening and baseline data assessment.
T2: Assessment after of Step 1 and before beginning of Step 2 intervention; for families randomised to Waiting Control in the moderate group, there are two T2 assessments: one after the three-month waiting period and one after the self-help programme.
T3: Assessment after Step 2 intervention.
T4: Follow-up assessment three months after the end of Step 2 intervention.
TASH: Step 1 treatment arm of the moderate group receiving telephone-assisted self-help for parents and teachers.
Waiting Control: Step 1 treatment arm of the moderate group randomised to waitlist control group.
